# Molecular Docking and Preclinical Study of Five-Membered S,S-Palladaheterocycle as Hepatoprotective Agent

**DOI:** 10.15171/apb.2019.079

**Published:** 2019-10-24

**Authors:** Nail Salavatovich Akhmadiev, Albina Midkhatovna Galimova, Vnira Rakhimovna Akhmetova, Veronika Radievna Khairullina, Rozaliia Akramovna Galimova, Eduard Feliksovich Agletdinov, Askhat Gabdrahmanovich Ibragimov, Valery Alekseevich Kataev

**Affiliations:** ^1^Institute of Petrochemistry and Catalysis, Russian Academy of Sciences, 141 Prospekt Oktyabrya, 450075 Ufa, Russia.; ^2^Bashkir State Medical University, 3 Lenin Str., 450008 Ufa, Russia.; ^3^Bashkir State University, 32 Zaki Validi Str., 450076 Ufa, Russia.

**Keywords:** Cytochrome P450 CYP2E1, Hepatoprotective activity, Liver, Molecular docking simulation, Palladaheterocycle, Preclinical study

## Abstract

***Purpose:*** In order to investigate mechanisms underlying the hepatoprotective action of S,Spalladaheterocycle, inhibition of cytochromes P450 has been modeled by molecular docking of four palladaheterocycle stereoisomers to the active sites of an enzymatic oxidase system. To obtain a deeper insight into biochemical aspects providing a basis for the therapeutic effects of five-membered palladacycles (as mixture of stereoisomers), a number of preclinical trials has been conducted

***Methods:*** 2D and 3D structures of palladaheterocycle stereoisomers were obtained via converting into SDF files by means of software MarvinSketch. Binding of palladaheterocycle at the active sites of cytochromes P450 2E1 and P450 2C9 has been studied by molecular docking using LeadIT 2.3.2. Hepatoprotective activity of palladaheterocycle at 2.5, 25 and 250 mg/kg doses has been studied based on a model of acute intoxication by CCl_4_ using *in vivo* methods.

***Results:*** By molecular docking it was identify amino acid fragments responsible for binding with palladacyclic isomers. The tested compound is comparable, in terms of its activity to the hepatoprotective drug SAM according to the *in vivo* and *in vitro* experiments such as animal survival data, the efficiency of correction of the cytolytic syndrome, the liver excretory function, carbohydrate, protein and lipid metabolism, and the correction efficiency of the liver antitoxic function (the latter has been determined based on the results of a hexobarbital control experiment).

***Conclusion:*** Taking into account results obtained *in vivo, in vitro* and in silico, it can be concluded that the five-membered S,S-palladaheterocycle effectively protect the liver against acute damage caused by CCl_4_ , via activation of catalase and glucuronyltransferase, as well as via inhibition of the oxidative stress enzymes.

## Introduction

Problems associated with hepatic disorders that lead to disruption of vital metabolic processes^1,2^ are triggered by hepatitis or HIV viruses, mycotic infections, hemochromatosis, schistosomiasis, as well as by obesity and intoxication (alcohol, drugs, chemicals).^3-5^ To restore of metabolic processes in the liver cells, various hepatoprotective agents with multidirectional bioregulation are traditionally utilized.^6^

A modern approach is based on implementation of biochemical processes via binding drug molecules to a number of enzymatic systems, including the cytochrome P450 system and the other microsomal enzymes.^7,8^

A noteworthy cluster of drugs based on enzyme-ligand interactions includes sulfur-containing derivatives (sulfides, sulfoxides, sulfones, thionic salts) and nitrogen-containing derivative (amines, amino acids, azaheterocycles), said cluster being represented by a wide range of pharmaceuticals and natural products.^9-11^ Amongst the sulfur-containing and nitrogen-containing commercialized hepatoprotector drugs, a special reference should be made to malotilate,^12^ SAM,^13^ thiotriazolin,^14^ thioctic acid and taurine.^15,16^

Another innovative cluster of drugs is represented by metal complexes generated based on a concept of modeling the metal-enzyme interaction sites, in where interactions often involve metal-thiolate or metal-amine bonds.^17^ The above concept is illustrated by a synthetic hepatoprotective drug Antral based on an aluminum complex with mefenamic acid - tris[N(2,3-dimethylphenyl)anthranilate] aluminum.^18^ Similarly, a copper complex of Baikalin demonstrates superior hepatoprotective properties, as its activity exceeds the same possessed by the parent flavonoid ligand.^19^ A number of studies exist demonstrating that trivalent chromium has protective activity against acute lethal liver damage induced by carbon tetrachloride, whose activity is based on detoxifying trichloromethyl (•CCl_3_) radicals.^20^

**Figure 1:**
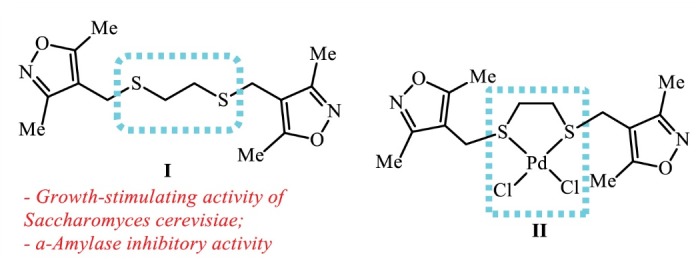
The structure of the sulfanyl-substituted 3,5-dimethylisoxazole **I** and of its derivative - palladium(II) containing heterocycle **II**.

Recently, we have reported the synthesis of sulfanyl-substituted *bis*(3,5-dimethylisoxazole) **I**^21^ and of its adduct with PdCl_2_ (Figure 1), which both have hepatoprotective activity and belong to a group of virtually non-toxic substances (class IV).^22,23^

Here by, we report the results of further in-depth studies assessing the therapeutic potential of the isoxazolylmethylsulphanyl substituted palladaheterocycle **II** as a hepatoprotector.

## Materials and Methods

### Chemistry assay and materials

^1^Н, ^13^С NMR Spectra were acquired on a Bruker Ascend III HD 500 spectrometer (500, 125 MHz, respectively) in DMSO-d_6_, using TMS as internal standard. *Сis*-S,S-dichloride-1,6-(3,5-dimethylisoxazol-4-yl)-2,5-dithiahexane palladium(II) – compound **II** has been synthesized according to a published procedure.^24^ The characteristics of palladacycle **II** has been identical to the literature data.^24^

### Molecular docking

3D Structures of all palladacycle enantiomers **IIa**,**a**′,**b**,**b**′ were obtained via converting into the SDF files using the MarvinSketch software (version 15.8.17). Henceforward, the preliminary molecular docking calculations of all stereoisomers **IIa**,**a**′,**b**,**b**′ were performed (see Supplementary file 1, Tables S1-S2). The cytochromes P450 2E1 and P450 2C9 were used for evaluation the antitoxic activity of the compounds **IIa**,**a**′,**b**,**b**′.To simulate these enzymes, we used macromolecules with PDB ID 3gph and 4nz2, respectively, taken from the Protein Data Bank.^25^

Molecular docking was performed with the LeadIT software package (version 2.3.2, BioSolveIT GmbH, St Augustin, Germany).^26^ FlexX algorithm followed by HYDE algorithm was used consistently to score, rank and generate the binding poses of four stereoisomers **IIa**,**a**′,**b**,**b**′ (see Supplementary file 1 , Figure S1) interacting with cytochromes P450 2E1 and P450 2C9.^26-28^ Both algorithms consider ligand flexibility by varying potential bioactive conformations of the ligands at the active sites while making the proteins rigid.

Molecular docking was performed with the following parameters: 1) default general docking information; 2) base placement using triangle matching; 3) scoring of full score contribution and threshold of 0.3 and No score contribution and threshold of 0.7; 4) default docking details values of 200 for both the maximum number of solutions per iteration and the maximum number of solutions per fragmentation.

Chemical parameters of clash handling values for protein ligand clashes with maximal allowed overlap volume 5.0 Å^3^ and intra-ligand clashes with clash factor of 0.6 and considering the hydrogen in internal clash tests. Best 10 poses according to the FlexX score were stored for rescoring with HYDE. The interactions between the four enantiomers **IIa**,**a**′,**b**,**b**′ and two mentioned receptors as docking complexes were analyzed by pose-view of LeadIT. For docking studies, description files for both receptors were prepared by using LeadIT graphic interface. Active sites of both proteins were defined by selecting the appropriate amino acid residues. Active sites of both enzymes thus includes protein residues at around 14 Å radius centered on the center of mass of the reference ligand (omega-imidazolyl-decanoic acid for P450 2E1 and (2R)-N-{4-[(3-bromophenyl)sulfonyl]-2-chlorophenyl}-3,3,3-trifluoro-2-hydroxy-2-methylpropanamide for P450 2C9, respectively).^25^

All the docking poses and interacting studies were visualized using Discovery Studio Visualizer software (version 16.1.0.15350).

To test the applicability of the LeadIT 2.3.2 program to modeling macromolecules with the PDB accession codes 3gph and 4nz2, we preliminarily used the molecular redoсking of their native ligand structures, for which coordinates of their locations in the active sites of the cytochromes are known from the PDB Protein Data Bank.^25^ Root-mean-square deviation values deduced from the calculated coordinations of these compounds with their native positions in the active sites were in the range of 1.5-2.1 Å. This indicates the applicability of the evaluating functions of the LeadIT 2.3.2 program to simulating inhibitors for cytochromes P450 2E1 and P450 2C9.

### Biological assay

120 White outbred rats of the same age, quarantined at a vivarium, with no external signs of any disease have been used in our investigation. Animals were kept in standard vivarium conditions on a normal diet.^29^ The experiments have been carried out in accordance with the “Rules for carrying out work using experimental animals” (Appendix to the Order of the Public Health Ministry of the USSR No. 755 of 12.08.1977), the “Directive 2010/63/EU of the European parliament and of the Council on the protection of animals used for scientific purposes” of 22 September 2010 and the requirements of the Order of the Public Health Ministry of the Russian Federation No. 199 of 01 April 2016 “On the approval of the rules of proper laboratory practice”.

Hepatoprotective activity has been studied in the model of acute toxic hepatitis caused by the introduction of carbon tetrachloride.^30^ The tetrachloromethane (CCl_4_) in 50% solution of olive oil has been injected intraperitoneally once at a dose of 0.3 mL/kg.

The test compound **II** has been administered intraperitoneally (ip) at doses of 2.5, 25 and 250 mg/kg 1 h prior to administration of CCl_4_. A pharmacologically known hepatoprotective drug – S-adenosylmethionine namely SAM **III**(see Supplementary file 1 , Figure S2) has been used as a control, upon intraperitoneal administration at a dose of 25 mg/kg.

A animals in the control group have received an equivalent volume of physiological sodium chloride solution 1 h prior to the administration of CCl_4_. Animals in an intact group have been injected intraperitoneally with an equivalent volume of physiological sodium chloride solution.

The studies have been carried out in two stages: *in vivo* (n = 60) and after decapitation of animals under ether anesthesia a day after the administration of the **II** and SAM (n = 60). One day after administering the compounds **II** and **III** to the groups of animals as listed above (n = 60), rodent euthanasia has been performed by decapitation under ether anesthesia, whereupon biological material has been collected for study.

Severity of inflammatory processes in liver tissue has been assessed by estimating a relative weight of the liver (defined as a ratio of the liver weight in mg to the total body weight in g).

Histological structure has been studied in liver tissue specimen stained by hematoxylin-eosin and the number of necrotic hepatocytes (having nuclei in the states of pyknosis, lysis or otherwise non-nuclear) provided as 2500 cells in 40 fields of vision has been determined morphometrically. Morphometric determination has been also used to determine the sizes of the nucleus and the cytoplasm, as well as the ratio thereof. The index of histological activity (IGA) has been determined by standard methods on a histological complex MICROM (“Carl Zeiss”, Germany) histological complex. A four-point system has been utilized for assessing indicators of protein dystrophy, inflammatory infiltration and hyaline-droplet dystrophy.

#### Determination of the degree ofadipose liver dystrophy

The study has been carried out histochemically using coloration by Sudan IV. Sections have been prepared on a freezing microtome from pieces of liver tissue fixed in 10% neutral buffered formalin. For a semi-quantitative assessment of lipid content, a five-point scale has been used as follows:

1. Minimal degree of obesity. Hepatocytes with fatty inclusions locate only on the periphery of the lobule in the region of the triad;
2. Weak degree. Hepatocytes containing lipids occupy approximately 1/4-1/3 of the length of the hepatic beams in the periportal zone;
3. Moderate degree. Uniform hepatocytes occupy 1/3-1/4 of the length of the hepatic beams along the periphery of the lobule;
4. High degree. Hepatocytes with fatty drops occupy 1/2-2/3 of the length of the hepatic beams;
5. Maximum degree. Steatosis extends to the entire hepatic lobule.

In order to obtain a differential diagnosis for the pathological syndromes, to evaluate the efficiency- and to elucidate the mechanism of action of potential hepatoprotective agents in serum, an activity assessment has been performed to include a number of indicator enzymes involved in development of the cytolytic syndrome, including aminotransferases (ALT-UV-Novo, AST-UV-Novo), γ-glutamyltransferase (Gamma-GT-Novo), and lactate dehydrogenase (LDH-UV-Novo); as well as the enzymes known as the markers for cholestasis, such as alkaline phosphatase (Alkaline phosphatase-Novo). A number of components has been quantitatively determined in serum, including total protein, albumins, glucose, triglycerides, cholesterol, and urea. A content of total bilirubin and direct bilirubin levels have also been determined. The bilirubin glucuronidation coefficient has been calculated as the ratio of the concentrations of glucuronic acid (direct) and total bilirubin-bound. Based on the results of these studies, an ability of hepatocyte enzymes to catalyze conjugation reactions can be evaluated. The reagent kits provided by ZAO Vector-Best (Russian Federation) have been used. The determination analysis has been conducted by a biochemical analyzer Bialab-100.

To evaluate the intensity of lipid peroxidation (LPO) and the activity of an antioxidant system in liver homogenates, a content of malonic dialdehyde has been determined by a reaction with thiobarbituric acid. The rate of antioxidant liver protection has been assessed by a catalase activity assay.^31^

At the second stage of the experiment (n = 60), survival rate (in percent, %) has been evaluated on the tenth day of observations. To assess antitoxic functions of the liver, a hexobarbital (hexenal) test has been performed (60 mg/kg, intraperitoneally). Latency and duration of anesthesia (hexenal sleep) have been determined.

### Statistical analysis

Conventional methods of descriptive statistics have been used to process the results expressed as the arithmetic mean (M) and as its standard error (m). Statistically significant differences have been determined using non-parametric statistical tests, such as Mann-Whitney (U) test and Wald-Wolfowitz (WW) test, as well as a parametric Student’s test (t-test) (after checking selected samples for the normality of distribution (Shapiro-Wilks criterion) and equality (Levine’s criterion)). Statistical interrelations have been studied using nonparametric correlation analysis, whereby the Spearman’s correlation coefficients for ranks have been calculated using software known in the art, hereby, Statistica 8.0 for Windows.^32^

## Results and Discussion

For identify of the biochemical aspects of the hepatoprotective action of the palladaheterocycle **II**, we have performed a number of preclinical studies of compound **II** as the diastereomeric mixture showing high activity. Additionally, we have optimized the position of palladaheterocycle **II** within the active site of the cytochrome P450 (CYP2E1, CYP2C9) by estimating the interaction energy of the four stereoisomeric ligands **IIa**,**a**′,**b**,**b**′ with the enzyme by means of molecular docking.

### Chemistry

Sulfinyl substituted bis(3,5-dimethylisoxazole) **I** synthesized by a one-pot method^21^ possesses multifunctional biological properties, including an inhibitory activity against α-amylase,^33^ a growth stimulating activity against *Saccharomyces cerevisiae*,^34^ as well as the hepatoprotective activity. However, a metal adduct of the compound **I** with PdCl_2_, being in the form of a five-membered S,S-palladaheterocycle **II** (Figure 1), had considerably improved hepatoprotective activity. Mentioned activity has been demonstrated by *in vivo* tests, while using the drug SAM (Heptral) as a control.^22^

By dynamic NMR ^1^H it has been established that the palladacycle **II** in solution occurs as a mixture of two diastereomers, namely *anti*-**IIa** and *syn*-**IIb**, in a 2:1 ratio. Reaction rate constants for the conversion between *anti*-**IIa** and *syn*-**IIa**were equal to 9.0 s^-1^ (293 K), 20.2 s^-1^ (303 K).^24^

The ligand has C2 symmetry with identical isoxazolyl-CH_2_-moieties about both sulfur atoms. As a result, *anti*-**IIa** and *syn*-**IIb** diastereoisomers are characteristic of the compound **II**. When provided as an enantiomeric mixture, the molecule **II** has four stereoisomers, namely RR-**IIa** or SS-**IIa**′ and SR-**IIb** or RS-**IIb**′ (see Figure S1, Supplementary file 1), wherein, according to the data obtained by NMR ^1^H, an *anti*-isomer is more preferable, which is consistent with the literature data.^35,36^ Interconversion between the *anti*-**IIa** and *syn*-**IIb** isomers has been observed in solution. Apparently, this dynamic isomerization increases probability of adsorption interaction with biological molecules. In view of this flexibility, the compound **II** is capable of adapting itself to the active centers of the enzymes.

### Molecular docking

Molecular pathways underlying the acute pathogenesis of the liver are associated with complex mechanism that includes processes of oxidative stress, apoptosis, inflammation and necrosis. Carbon tetrachloride (CCl_4_) is a hepatotoxin commonly used in experimental animal models due to the similar molecular mechanisms of the chemical damage of human and animal livers. As shown, CCl_4_ is metabolized by the cytochrome P450 (CYP2E1) system and converted to trichloromethyl (•CCl_3_) and trichloromethyl peroxyl (•CCl_3_OO) radicals. Then these free radicals induce the peroxide oxidation of membrane lipids.^1,37^ Disturbance of hepatocellular function leads to accumulation of bile acids that cause additional toxicity and cytotoxicity.^38^ The CCl_4_-induced inflammation leads to the acute liver damage accompanied by formation of flash mediators, including tumor necrosis factor-alpha TNF-α, inducible nitric oxide synthase and cyclooxygenase-2, via activation of the nuclear factor-kappa B (NF-κB) in the liver of animals.^39^

However, cytochrome P450 2E1 is not the sole enzyme involved in the metabolism of xenobiotics. Indeed, P450 2C9 is another enzyme from the cytochrome P450 family that plays an important role in the oxidation of xenobiotic and endogenous compounds including drugs. The concentration of P450 2C9 is about 18% of cytochrome P450 in liver microsomes.^40^ As reported,^41^ cytochrome P450 of the CYP2C9 family metabolizes drugs and important endogenous compounds (e.g., serotonin). Additionally, it has the epoxygenase activity and metabolizes polyunsaturated fatty acids into a wide range of biologically active products. Hence, the study of both cytochromes should promote a deeper insight into molecular interactions and provide the estimates of the antitoxic activity of the compound **II** as an inhibitor of cytochromes P450 2E1 and P450 2C9.

Using the LeadIT program, we have performed flexible molecular docking of four isomers **IIa**,**a**′,**b**,**b**′ at the active sites of cytochromes P450 2E1 and P450 2C9. These calculations are based on the pre-selected models of the Human cytochromes 3gph and 4nz2 (PDB accession codes). Such an approach is justified due to a rather high similarity between the active sites in the related enzymes of humans, mice and rats.

**Figure 2:**
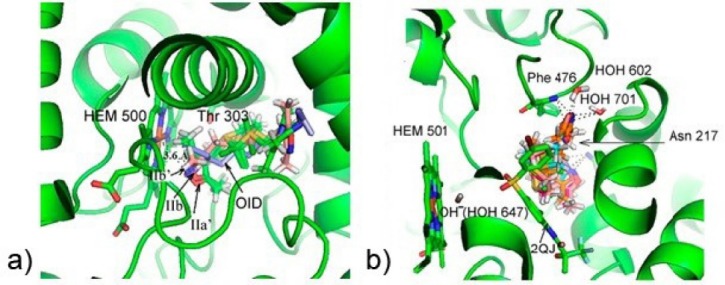
**a)** Positioning of reference ligand with code OID and ligands **IIa′**, **IIb**, **IIb′** in the active center of the protein with the 3gph code (model of the enzyme cytochromes P450 2E1); **b)** Positioning of reference ligand with code 2QJ and ligands **IIa**, **IIa′**, **IIb**, **IIb′** in the active center of the protein with the 4nz2 PDB accession code (a model of the enzyme cytochromes P450 2С9).

Docking interactions between the amino acids at the binding sites of these cytochromes and four enantiomers **IIa**,**a**′,**b**,**b**′ are presented in Table 1 and on Figure 2a,b.

**Table 1:** Hydrogen bonding and hydrophobic interactions ofthefour palladaheterocycle enantiomers **IIa**,**a**′,**b**,**b**′with cytochromes P450 2E1 and P450 2C9

**Diastereomers**	**Cytochrome P450 2E1**		
	**E** **_bind_** **, kJ/mol**	**Hydrogen bonding interaction**	**Non bonded interaction**
IIa	Sterically not complementary
IIb	-37 ± 3.0	**‒**	**Phe106,**Ile 115, Phe 116, Phe 203, Asn 204, Asn 206, **Phe207**, Leu 210, Val 239, **Phe298**, **Ala 299**, Clu 302, Thr 303, Leu 363, **Val 364**, Cys 437, **Leu 368**, **Phe478**, Hem 500
IIb′	-26 ± 2.5		
IIa′	-31 ± 3.0		
**Diastereomers**	**Cytochrome P450 2C9**		
	**E ** **_bind_** **, kJ/mol**	**Hydrogen bonding interaction**	**Non bonded interaction**
IIa	-38 ± 3.0	Phe 476, HOH 602, HOH 701	Phe 100^π- π^, **Leu 102**, **Ala 103**, Phe 114, **Ile 205**, **Leu 208**, Ser 209, Ile 213, Asn 217, Leu362, Ser 365, Leu 366, **Pro367**, Gly 475, **Ala 477**
IIb	-42 ± 3.0		
IIb′	-42 ± 3.5		
IIa′	-35 ± 3.0		

Amino acid residues of the proteins involved in hydrophobic interactions with ligand molecules are shown in bold.

The results of the molecular docking (Table 1) show that the enantiomers**IIa**,**a**′,**b**,**b**′in the active centers of both enzymes are positioned in different modes.

In the active site of cytochrome P450 2E1, three enantiomers (SR **IIb**, SS **IIa**′, RS **IIb**′), except of RR **IIa**, fit in the same spatial region as the reference ligand ω-imidazolyl-decanoic acid and, therefore, form a single cluster. Enantiomer RR **IIa** is not complementary with the active site of the enzyme. Upon participation of the nitrogen atom of the oxazole cycle, three of them are able to form a coordination bond with the iron located in heme. Van der Waals and hydrophobic interactions with the neighboring amino acids additionally stabilize the three enantiomers in the P450 2E1 active site (see Table 1). The interactions of three enantiomers SR **IIb,** SS **IIa**′, RS **IIb**′ with the active center of P450 2E1 are moderate according to the HYDE scoring function built in the LeadIT program. As follows from the free binding energies, enantiomer SR **IIb** should most efficiently interact with this protein.

Cytochrome P450 2C9 has a significantly larger cavity available for binding in contrast to cytochrome P450 2E1. This stipulates a fundamental difference in positioning of all four enantiomers of palladaheterocycle **II** as compared to enzyme Р450 2Е1. In the case of cytochrome P450 2C9, the ligand molecules are unable to replace the hydroxide anion from the internal coordination sphere of the heme iron. As a result, all four enantiomers **IIa**,**a**′,**b**,**b**′ are located in the external coordination sphere of this complex. All of them form a single cluster in the active site of the protein under study. However, they are positioned in another spatial region due to their structural differences from the reference ligand. Positions of all four enantiomers of compound **II** at the active site of the P450 2C9 enzyme are stabilized by hydrogen bonds with Phe 476, HOH 602, HOH 701, π-π stacking interactions with Phe 100, van der Waals and hydrophobic interactions primarily with neighboring non-polar amino acid residues. Based on the evaluative HYDE function, all four enantiomers are able to effectively bind to cytochrome P450 2C9. However, the preference should be given to the enantiomers SR **IIb** and RS **IIb**′.

### Preclinical study

Biotesting has been carried out using standard medication with SAM – S-adenosylmethionine **III**(see Supplementary file 1, Figure S2), which is known to be involved in processes of transmethylation, transsulfuration pathway and aminopropylation. The compound **II** has a number of fragments in its structure, namely, methyl groups, sulfur atoms and nitrogenous heterocycles, similar to that in SAM.

#### Evaluation of survival in induced acute liver pathology

In the modeling studies of toxic hepatitis using SAM as a control, upon administration of the compound **II** to the experimental groups a significant reduction in animal lethality has been observed (see Supplementary file 1, Table S3). Thus, an analysis of survival rates demonstrated that hepatoprotective properties of the **II** are comparable to that of the control drug SAM**III**.

#### Hexobarbital test on the model of acute liver pathology

**Figure 3:**
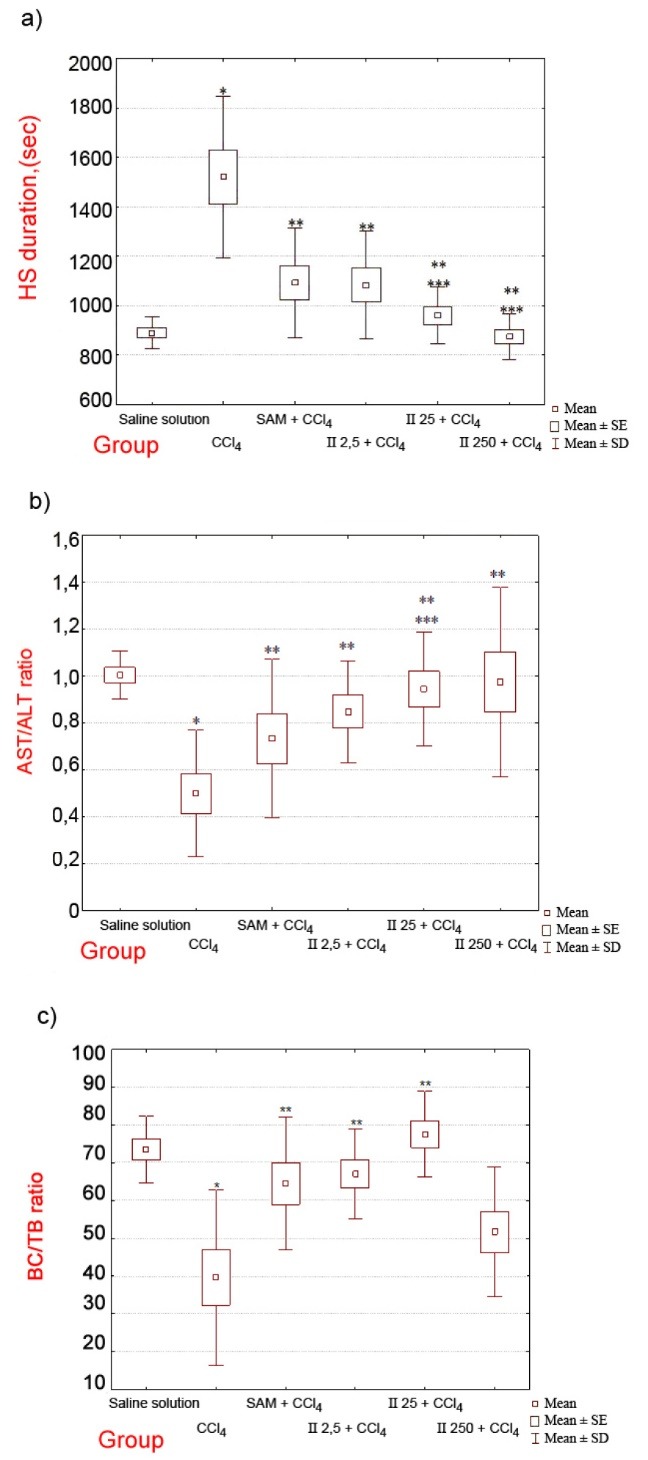
**a)**An impact of the palladaheterocycle **II** on duration of hexobarbital sleep (HS); **b)** an impact of the palladaheterocycle **II** on changes in AST/ALT ratio upon modeling the acute toxic hepatitis (Note: * Statistically significant differences from the indices of a group of intact animals; ** Statistically significant differences from the indicators of the group “CCl_4_”; *** Statistically significant differences from the indicators of the group “CCl_4_ + SAM”); **c)**an impact of the palladaheterocycle **II** on changes in the bilirubin glucuronic nucleation rate in case of modeling the acute toxic hepatitis (Note: Conjugated bilirubin (BC); Total bilirubin (TB); * Statistically significant differences from the indices of a group of intact animals; ** Statistically significant differences from the indicators of the group “CCl_4_”).

A hepatoprotective effect of the palladaheterocycle **II** has been demonstrated while assessing the antitoxic function of the liver according to a hexobarbital (hexenal) test (Figure 3a).

Administration of CCl_4_ to the animals causes an increase in duration of hexobarbital induced sleep (HIS), which is indicative of the metabolic rate of hexobarbital reduced under the effect of the cytochrome P-450-dependent monooxygenase system in hepatocytes, the latter being impacted by the toxic effect of CCl_4_. Nevertheless, preliminary administration of the compound **II** resulted in reduction of HIS duration. HIS duration in the group that received the palladaheterocycle **II** at a dose of 2.5 mg/kg has been comparable to the same in the group of rats that received the control compound SAM. In groups that received the S,S-palladaheterocycle **II** at the doses of 25 mg/kg and 250 mg/kg, a statistically significant reduction in HIS duration has been observed, as compared to the corresponding index value obtained for the group receiving the control compound **III**.

Thus, according to the results of the hexobarbital test conducted on the model of acute toxic hepatitis induced by administration of CCl_4_, the compound **II** demonstrates a greater effectiveness with regard to preventing violation of the antitoxic function in the liver tissue, as compared to the control drug SAM.

#### Changes in body weight and liver weight in induced acute liver pathology

In experimental studies using the models of hepatic pathology, the most important integral indicators demonstrative of general condition of experimental animals and of the state of their hepatobiliary system are the changes in the body weight of an animal and the changes in relative mass of the liver organ, accordingly^29^

Tetrachloromethane when administered to animals had a tendency to decrease the body weight, but its value did not reach statistical significance (Table 2).

**Table 2:** An impact of the S,S-palladaheterocycle **II** on the liver mass index in animals induced toxic hepatitis

**Group**	**Index**		
	**Rat weight, g**	**Liver weight, mg**	**Relative liver mass**
Normal saline solution 0.2 mL/kg (intact group)	195.46 ± 4.78	5748.60 ± 316.36	29.37 ± 1.41
CCl_4_ 0.3 mL/kg (control group)	189.84 ± 3.93	8836.00 ± 335.27^a^	46.68 ± 1.89^a^
SAM 25 mg/kg i.p. + CCl_4_ 0.3 mg/kg	203.05 ± 3.07	8239.40 ± 288.47	40.56 ± 1.20 ^b^
**II**2.5 mg/kg i.p. + CCl_4_ 0.3 mg/kg	197.04 ± 4.54	8218.50 ± 295.65	41.87 ± 1.69
**II**25 mg/kg i.p. + CCl_4_ 0.3 mg/kg	197.97 ± 4.33	7271.30 ± 408.89^b,c^	36.70 ± 1.77^b,c^
**II** 250 mg/kg i.p. + CCl_4_ 0.3 mg/kg	188.15 ± 3.66	6667.10 ± 253.75^b,c^	35.53 ± 1.48^b,c^

^a^ Statistically significant differences from the indices of a group of intact animals; ^b^ statistically significant differences from the indicators of the group “CCl_4_”; ^c^statistically significant differences from the indicators of the group “CCl_4_ + SAM”.

It is known that significant decrease in body weight of the occurs at later intoxication stages during the development of CCl_4_-induced toxic hepatitis.^29^ Another characteristic manifestation of acute toxic hepatitis that was observed is a significant increase in the liver weight, evidently due to inflammatory changes in the organ (Table 2). Preliminary administration of SAM slightly reduced the increase in the liver weight. The compound **II** dose-dependently reduced the weight of the organ which also indicates its hepatoprotective effect.

#### Activity of hepatic enzymes in serum

During the course of a study on activity of enzymes – indicators of the cytolytic syndrome in blood serum (Table 3), we have observed sharp increase in activity of transaminases and γ-glutamyltransferase after administration of CCl_4_.

**Table 3:** An impact of the palladaheterocycle **II** on an activity of hepatic enzymes in the serum in the induced toxic hepatitis

**Group**	**Index**
	**ALAT, IU/L**	**AspAT, IU/L**	**GGT, IU/L**	**ALP, u/L**
Intact group CCl_4_	40.46 ± 3.59	40.10 ± 3.24	11.32 ± 1.36	395.12 ± 34.18
	704.10 ± 110.40^a^	330.75 ± 73.47^a^	34.48 ± 4,24^a^	1190.76 ± 208.75^a^
SAM 25 mg/kg + CCl_4_	362.90 ± 75.48^b^	242.14 ± 49.59^b^	16.31 ± 3.33^b^	569.77 ± 100.31^b^
**II** 2.5 mg/kg + CCl_4_	313.88 ± 54.99^b^	248.94 ± 38.34^b^	12.52 ± 2.42^b^	364.15 ± 77.23^b^
**II** 25 mg/kg + CCl_4_	236.62 ± 47.13^b^	218.35 ± 45.43^b^	14.66 ± 2.40^b^	514.95 ± 67.07^b^
**II** 250 mg/kg + CCl_4_	215.39 ± 43.67^b^	172.23 ± 10.73^b^	12.71 ± 2.81^b^	413.04 ± 74.60^b^

^a^ Statistically significant differences from the indices of a group of intact animals; ^b^ statistically significant differences from the indicators of the group “CCl_4_”.

Preliminary administration of SAM and the S,S-palladaheterocycle **II** at each studied dose reduced the activity of the indicator enzymes of cytolytic syndrome in the blood serum. At the same time this effect was more pronounced in groups of animals that received the compound **II**. However, despite the obvious trend, there were no statistically significant differences between the group pre-treated with SAM and the experimental groups receiving palladaheterocycle **II**.

The indicator for toxic liver damage and the development of cytolytic syndrome is considered to be not only the increase in activity of enzymes responsible for cytolysis in the serum, but also a relatively high level of ALT (alanine transaminase) activity. The AST/ALT activity ratio, also known as de Ritis coefficient in the group of animals affected by “CCl_4_” has been significantly reduced compared to the corresponding index for intact animals (Figure 3b).

Preliminary administration of both SAM and the compound **II** at all studied doses has had a restrictive effect on reduction of the de Ritis coefficient values. In this regard, the most effective doses have been 25 mg/kg (giving rise to statistically significant differences in the group “CCl_4_ + SAM”) and 250 mg/kg (being the closest to the normal mean value of the de Ritis coefficient). Thus, preliminary administration of the palladaheterocycle **II** has had a restrictive effect on the CCl_4_-induced cytolysis of hepatocytes. An impact level of the compound **II**, in view of its hepatoprotective characteristics, is comparable, at a dose of 2.5 mg/kg, to the impact level of SAM and exceeds the effect obtainable by administration of SAM at doses of 25 and 250 mg/kg.

Similar conclusions can be drawn from the analysis of the cholestasis value, i.e. alkaline phosphatase value (Table 3). Preliminary administration of the compound **II** inhibits the CCl_4_-induced increase in the serum activity indicating a positive effect of palladaheterocycle **II** on the excretory function of the liver. A hepatoprotective effect was most significant at the dose of the compound **II**equal to 2.5 mg/kg.

#### Pigment exchange and excretory liver function

The study conducted on pigment metabolism, along with investigating the excretory function of the liver (as bilirubin being a pigment of the bile), enables assessment of activity of the key enzymes, such as UDP-glucuronyltransferase, in the second phase of xenobiotics biotransformation.

Administration of CCl_4_ to the experimental animals has been accompanied by a statistically significant increase of a bilirubin content in serum (see Table S4, Supplementary file 1).

Predominance of unconjugated bilirubin in the serum indicates the impairment in the transport from blood and conjugation of the pigment in hepatocytes. The medications SAM and the palladaheterocycle **II** at each studied doses had reduced CCl_4_-induced hyperbilirubinemia to the similar extent, but despite a decrease in the average values of this indicator no statistically significant differences have been detected. Despite a relatively weak effect on total bilirubin, palladacycle **II** significantly affected the activity of bilirubin conjugation in hepatocytes changing the ratio between conjugated and unconjugated pigment in the favor of the former (Figure 3c).

This effect has been more pronounced in comparison to that attained upon administration of SAM, whereas the most pronounced effect has been attained upon preliminary administration of the compound **II** at a dose of 25 mg/kg (equivalent to that of the control drug). Mentioned conditions have been shown to cause changes in the bilirubin glucuronic nucleation rate factor (Figure 3c). The results of the studies conducted hereby provide strong evidence on an ability of hepatocyte enzymes to catalyze conjugation reactions; therefore, it appears justified that administration of the **II** at a dose of 25 mg/kg prevents bilirubin conjugation in acute toxic hepatitis.

#### Content of proteins, lipids, carbohydrates in bloodstream

Since the liver plays an important role in metabolism of proteins, lipids and carbohydrates, evaluation of its protein-synthetic function and of the key parameters related to carbohydrate-, lipid- and nitrogen metabolism, in laboratory conditions, is an inevitable stage in assessment of the hepatoprotective action of drugs, along with the studies concerning antitoxic and excretory functions of the liver.

Despite a clearly pronounced tendency to develop hypoproteinemia upon administration of CCl_4_, statistical analysis of data has not confirmed a decrease in the level of total protein in serum. Probably, a longer temporal interval is required to develop violations in the protein’s function. If we analyze the data thus obtained based on the average values, one may observe that the maximum protective effect of the compound **II** in relation to the protein’s function emerges at the doses of 25 and 250 mg/kg (Table 4).

**Table 4:** An impact of the S,S-palladaheterocycle II on carbohydrate, lipid and nitrogen metabolism in the modeling of toxic hepatitis

**Group**	**Index**				
	**Total protein, g/L**	**Glucose, mmol/L**	**Triglycerides, mmol/L**	**Cholesterol, mmol/L**	**Urea, mmol/L**
Intact group	63.10 ± 2.16	4.22 ± 0.29	1.40 ± 0.12	3.36 ± 0.61	5.86 ± 0.54
CCl_4_	56.85 ± 2.66	3.02 ± 0.14^a^	2.17 ± 0.29^a^	7.47 ± 0.89^a^	2.15 ± 0.28^a^
SAM 25mg/kg + CCl_4_	61.30 ± 2.38	4.26 ± 0.18^b^	1.85 ± 0.25	5.30 ± 0.46^b^	3.79 ± 0.58^b^
**II** 2.5 mg/kg + CCl_4_	60.33 ± 3.01	4.51 ± 0.19^b^	1.38 ± 0.38^b^	5.03 ± 0.79^b^	3.74 ± 0.33^b^
**II** 25 mg/kg + CCl_4_	62.27 ± 2.62	4.37 ± 0.27^b^	1.66 ± 0.16^b^	5.25 ± 0.67^b^	3.26 ± 0.40^b^
**II** 250 mg/kg + CCl_4_	62.83 ± 1.95	4.16 ± 0.24^b^	1.42 ± 0.22^b^	4.55 ± 0.80^b^	4.53 ± 0.39^b^

^a^ Statistically significant differences from the indices of a group of intact animals; ^b^ Statistically significant differences from the indicators of the group “CCl_4_”.

SAM and the palladacycle **II** at all tested concentrations have demonstrated analogous efficiency rates with regard to the correction of CCl_4_-induced hypoglycemia, which is probably due to disruptions in the action of gluconeogenesis enzymes. Also hypertriglyceridemia and hypercholesterolemia, developed during the modeling of toxic hepatitis, have been significantly mitigated upon preliminary administration of both SAM and the compound **II** at all doses studied hereby (Table 4). Reduction in urea levels in blood serum with toxic hepatitis is most likely due to violations in fixation and neutralization of ammonia by the enzymes of the ornithine cycle. By analyzing the data obtained herewith, we can declare that preliminary administration of the palladaheterocycle **II** at all studied doses incurs protective effect on the liver function comparable to that incurred by SAM.

Thus, administration of the compound **II** prevents the development of disturbances in carbohydrate-, lipid- and nitrogen metabolism, which disturbances tend to develop upon modeling of acute toxic hepatitis. In such a case, the hepatoprotective efficiency of the S,S-palladaheterocycle **II** is generally comparable to that of the control drug.

#### Lipid peroxidation (LPO) and activity of the liver antioxidant system

This part of the study has been conducted bearing in mind an important role the antioxidant effect plays in mechanisms underlying the therapeutic effects exerted by hepatoprotectors. Additionally, one may suggest that the powerful antioxidant properties of the control drug (SAM), which are typically attributable to the presence of sulfur atoms and heterocyclic fragments in the molecule, are inherent also to the test compound, since the latter possesses structural characteristics similar to the control.^7^ The fact that liver pathology caused by hepatotoxin with pronounced prooxidant properties (carbon tetrachloride, CCl_4_) serves as a model for studying hepatoprotective properties in this study should also be taken into account. Extensive use of the oxidative stress inducers in experimental modeling of toxic hepatitis within a framework of investigating hepatoprotective effects of various compounds, is, in turn, attributable to a leading role played by the processes of free radical oxidation in liver pathology.^30^

Preliminary administration of both SAM and the palladaheterocycle **II** has resulted in the reduction of the oxidative stress level caused by administration of carbon tetrachloride. As can be observed from the object-related data in Table 5, the CCl_4_-induced toxic hepatitis is typically accompanied by the oxidative stress, whereupon the thiobarbituric acid reactive substances reach levels such, that the presence of mentioned reaction products can be recorded in both homogenates and animal sera.

**Table 5:** An impact of the palladaheterocycle II on severity of oxidative stress in induced toxic hepatitis

Group	Index		
	TBARS (liver), nmol/g	TBARS (blood serum), mkmol/mL	Catalase (liver), mcg/g
Intact group	0.45 ± 0.03	2.95 ± 0.14	67.26 ± 4.18
CCl_4_	1.72 ± 0.09^a^	11.16 ± 0.81^a^	25.43 ± 3.55^a^
SAM 25mg/kg + CCl_4_	1.25 ± 0.13^b^	7.89 ± 0.77^b^	37.33 ± 9.49
**II** 2.5 mg/kg + CCl_4_	1.25 ± 0.11^b^	8.32 ± 0.78^b^	64.59 ± 11.01^b^
**II** 25 mg/kg + CCl_4_	0.67 ± 0.06^b,c^	4.54 ± 0.38^b,c^	84.24 ± 7.99^b,c^
**II** 250 mg/kg + CCl_4_	0.76 ± 0.06^b,c^	4.88 ± 0.40^b,c^	90.75 ± 9.15^b,c^

^a^ Statistically significant differences from the indices of a group of intact animals; ^b^ Statistically significant differences from the indicators of the group “CCl_4_”; ^c^ Statistically significant differences from the indicators of the group “CCl_4_ + SAM”. TBA - RP - products reacting with thiobarbituric acid (TBA).

At the same time, decrease in activity of the one of the most important components of antioxidant protection, namely catalase, has been observed. The antioxidative effect of the palladaheterocycle **II** has been observed in whole blood samples and in blood serum.

It should be noted that the most pronounced antioxidant effect which exceeded the effectiveness of the control compound was registered at the doses of 25 and 250 mg/kg. Catalase activity in this case even exceeded the corresponding values observed in the group of intact animals. Thus, the data obtained demonstrate that the compound **II** is a promising candidate for a further study as a compound with antioxidant activity.

#### Histological processing of the liver

Administration of CCl_4_ causes severe structural changes emerging in the form of large-droplet hepatocyte dystrophy, lymphohistiocyte infiltration and violations of the liver structures.

Preliminary administration of the S,S-palladaheterocycle **II** and SAM results in less pronounced changes in the morphological structures of the liver, expressed as a decrease in inflammatory infiltration, necrosis of hepatocytes and reduction in the level of hepatocyte dystrophy.

Upon preliminary administration of the compound **II** and SAM, a significant decrease in the index of histological activity has been observed, in comparison to the data obtained for the control group of animals.

A minimal degree of fatty liver has been detected in the intact group of animals, whereupon hepatocytes with fatty inclusions have been found only on the periphery of the lobule in the triad region as follows: 1.20 ± 0.20 points (M ± m). When CCl_4_ has been administered to the animals of the corresponding experimental group, a high degree of fatty liver has been observed in most cases (n = 6), whereupon hepatocytes with fatty drops have been found to occupy 1/2–2/3 of the length of the hepatic beams. In four cases, the maximum degree of fatty degeneration has been observed, whereupon steatosis spread to the entire hepatic lobe had constituted 4.20 ± 0.25 points (M ± m). Preliminary administration of SAM and of the S,S-palladacycle **II** has limited the severity of fatty liver. In the group of animals previously treated with SAM at a dose of 25 mg/kg, the average index of fatty liver disease constituted 3.50 ± 0.27 points (moderate degree), and in groups of animals previously treated with the compound **II** at the doses of 2.5, 25 and 250 mg/kg, this index constituted 3.10 ± 0.31 (moderate degree), 2.50 ± 0.27 and 2.50 ± 0.17 (weak degree), respectively.

Thus, based on the model of acute hepatitis caused by carbon tetrachloride it has been shown that the novel, essentially nontoxic (class IV) compound S,S-palladaheterocycle **II** possesses hepatoprotective activity in laboratory animals (rats), at the doses of 2.5, 25 and 250 mg/kg administered intraperitoneally. Preclinical studies have demonstrated that administration of said compound **II** at the doses of 25 and 250 mg/kg exhibits maximum protective activity through activation of catalase and glucuronyl transferase has been observed along with the inhibition of enzymes of oxidative stress.

Molecular docking calculation confirms the inhibition of enzymes of oxidative stress. So, at the active site of cytochrome P450 2E1 three enantiomers (SR **IIb**, SS **IIa′**, RS **IIb′**), with the exception of RR **IIa**, located in the same spatial region as omega-imidazolyl-decanoic acid, selected as a reference ligand. Based on a comparative analysis of free binding energy values, the enantiomer SR **IIb** is most efficiently bound to enzyme. While, all four enantiomers, based on the estimated function of HYDE, are able to effectively bind to cytochrome P450 2C9. Moreover, the enantiomers of SR **IIb** and RS **IIb′** are the most preferred.

It has been established that S,S-palladaheterocycle **II** is comparable to the SAM comparison medication (S-adenosyl methionine, Heptral), in terms of animal survival rates, cytolytic syndrome correction efficiency, liver excretory functions, carbohydrate, protein and lipid metabolism. Moreover, the test compound is superior to the control drug in terms of its correction efficiency of the liver antitoxic function (hexobarbital test) and the antioxidant activity (catalase activity test).

## Ethical Issues

The study has been carried out according to standard ethical principles and relevant research regulations.

## Conflict of Interest

The authors declare that they have no conflict of interest.

## Acknowledgments

This work was financially partly supported by the Russian Foundation for Basic Research and Academy of Sciences of the Republic of Bashkortostan (Project numbers 17-43-020292 r_a and 18-29-09068 mk), Grants Council of the President of Russian Federation (grant NSh-5240.2018.3) and within the framework of the Project part of the State Assignment *AAAA-A19-119022290010-9*.

The structural studies of the compounds were performed with unique equipment in “Agidel” Collective Usage Centre.

## Supplementary files


Supplementary file 1 contains Figures S1-S2 and Tables S1-S4.
