# Supplementary material for: Molecular Docking and Preclinical Study of Five-Membered S,S-Palladaheterocycle as Hepatoprotective Agent
Source: Adv Pharm Bull. 2019 Oct 24;9(4):674–84. doi: 10.15171/apb.2019.079 (PMC6912183; doi:10.15171/apb.2019.079)
Supplement: Supplementary file 1 — contains Figures S1-S2 and Tables S1-S4. [file apb-9-674-s001.pdf]

Molecular Docking and Preclinical Study of Five-Membered S,S-Palladaheterocycle as Hepatoprotective Agent

Supplementary file 1

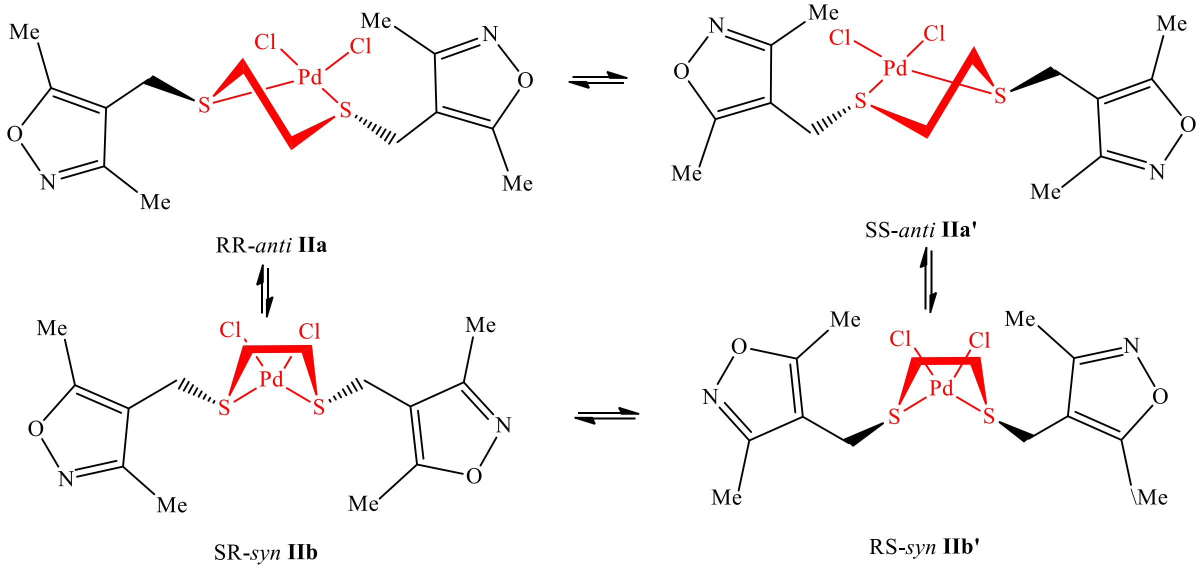

Figure S1. Diastereomers showing *syn*- and *anti*-isomerism of palladaheterocycle **II**.

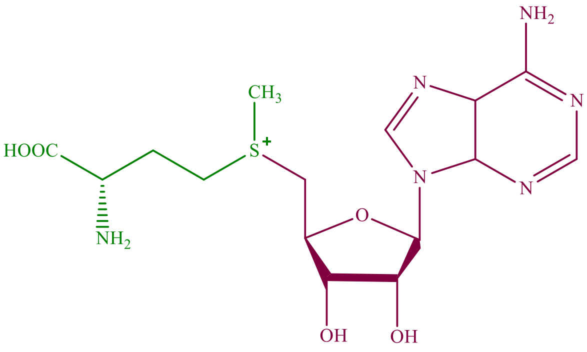

III

Figure S2. Structure of S-adenosylmethionine namely SAM **III**

Table S1. Molecular docking calculations of the enantiomers **IIa**,**a′**,**b**,**b′** in the active center of cytochrome P450 2E1

**IIa** with protein P450 2E1			RS	**IIb** with protein P450 2E1			SR	**IIb"** with protein P450 2E1			SS	**IIa"** with protein P450 2E1		
"-ΔG"	xmean-xi	(xmean-xi)^2			xmean-xi	(xmean-xi)^2								
3	2.4	5.76		44	7.2	51.84		31	4.9	24.01		36	5.1	26.01
2	3.4	11.56		40	3.2	10.24		29	2.9	8.41		35	4.1	16.81
8	-2.6	6.76		41	4.2	17.64		30	3.9	15.21		34	3.1	9.61
6	-0.6	0.36		38	1.2	1.44		30	3.9	15.21		32	1.1	1.21
3	2.4	5.76		30	-6.8	46.24		24	-2.1	4.41		30	-0.9	0.81
4	1.4	1.96		39	2.2	4.84		25	-1.1	1.21		31	0.1	0.01
9	-3.6	12.96		37	0.2	0.04		24	-2.1	4.41		29	-1.9	3.61
4	1.4	1.96		35	-1.8	3.24		23	-3.1	9.61		30	-0.9	0.81
6	-0.6	0.36		33	-3.8	14.44		22	-4.1	16.81		29	-1.9	3.61
9	-3.6	12.96		31	-5.8	33.64		23	-3.1	9.61		23	-7.9	62.41
no blunders identified				no blunders identified				no blunders identified				no blunders identified		
54		60.4		368				261				309		
5.4		5.04		36.8				26.1				30.9		
60.4	S=	2.244994432		183.6				108.9				124.9		
6.711111111	3S=	6.734983296		20.4				12.1				13.87778		
2.59058123				4.516636				3.478505				3.725289		
7.771743691	Dx=	1.604440837		13.54991				10.43552				11.17587		
2.26														
1.851422996				3.227926		37±3		2.486		26±2		2.66237		31±3

Table S2. Molecular docking calculations of the the enantiomers **IIa**,**a′**,**b**,**b′** in the active center of cytochrome P450 2C9

**IIa** with protein P450 2C9			RS	**IIb** with protein P450 2C9			SR	**IIb"** with protein P450 2C9			SS	**IIa"** with protein P450 2C9		
"-ΔG"	xmean-xi	(xmean-xi)^2			xmean-xi	(xmean-xi)^2								
47	9.1	82.81		48	6	36		54	12	144		37	1.9	3.61
40	2.1	4.41		44	2	4		45	3	9		35	-0.1	0.01
39	1.1	1.21		44	2	4		42	0	0		35	-0.1	0.01
39	1.1	1.21		40	-2	4		41	-1	1		34	-1.1	1.21
38	0.1	0.01		36	-6	36		40	-2	4		35	-0.1	0.01
37	-0.9	0.81		47	5	25		42	0	0		33	-2.1	4.41
39	1.1	1.21		39	-3	9		39	-3	9		25	-10.1	102.01
37	-0.9	0.81		43	1	1		42	0	0		44	8.9	79.21
34	-3.9	15.21		42	0	0		40	-2	4		36	0.9	0.81
32	-5.9	34.81		37	-5	25		35	-7	49		37	1.9	3.61
no blunders identified				no blunders identified				no blunders identified				no blunders identified		
382		142.5		420				420				351		
38.2		13.25		42				42				35.1		
142.5	S=	3.640054945		144				220				194.9		
15.83333333	3S=	10.92016483		16				24.44444				21.65556		
3.979112129				4				4.944132				4.653553		
11.93733639	Dx=	2.601455362		12				14.8324				13.96066		
2.26														
2.843770971				2.858699		42±3		3.533447		42±3.5		3.325777		35±3

Table S3. An impact of the palladaheterocycle **II** on a ten-day survival of animals in the modeling of toxic hepatitis

Group	$n_{initial\ amount}$	$n_{10\ day}$	Survival rates, %
Normal saline solution 0.2 mL/kg (intact group)	10	10	100
CCl_4 0.3 mL/kg (control group)	10	4	40
SAM **III** 25 mg/kg i.p. + CCl_4 0.3 mg/kg	10	7	70
**II** 2.5 mg/kg i.p. CCl_4 0.3 mg/kg	10	7	70
**II** 25 mg/kg i.p. CCl_4 0.3 mg/kg	10	8	80
**II** 250 mg/kg i.p. CCl_4 0.3 mg/kg	10	8	80

Note: $n_{initial\ amount}$ – initial number of animals in the group; $n_{10\ day.}$ – number of animals surviving for 10 days.

Table S4. An impact of the S,S-palladacycle **II** on pigment metabolism in induced toxic hepatitis

Group \ Index	Total bilirubin. mkmol/L	Conjugated bilirubin. mkmol/L	Unconjugated bilirubin. mkmol/L
Intact group CCl_4	10.87±0.73	7.92±0.50	2.95±0.45
	22.43±2.71[1]	8.69±1.43	13.74±2.38[1]
SAM 25mg/kg + CCl_4	17.91±1.02	11.92±0.91	6.99±1.22[2]
**II** 2.5 mg/kg + CCl_4	17.89±0.94	12.70±1.03[2]	6.19±0.75[2]
**II** 25 mg/kg + CCl_4	16.80±0.95	13.88±1.06[2]	3.92±0.65[2]
**II** 250 mg/kg + CCl_4	17.06±1.72	9.76±1.38	9.30±1.46

Note: [1] - statistically significant differences from the indices of a group of intact animals; [2] - statistically significant differences from the indices of the group "CCl_4"
